# Primitive reflexes in infants with cerebral palsy due to Congenital Zika Syndrome and its relationship with other motor features

**DOI:** 10.3389/fped.2025.1483959

**Published:** 2025-03-07

**Authors:** Leticia Serra, Débora Patrícia Rios, Mino Rios, Breno Lima de Almeida, Kelly de Souza Fernandes, Rita Lucena, Isadora Cristina de Siqueira

**Affiliations:** ^1^ Laboratorio de Investigação em Saúde Global e Doenças Negligenciadas, Instituto Gonçalo Moniz- Fundação Oswaldo Cruz, Salvador, Brazil; ^2^ Centro Estadual de Prevenção e Reabilitação da Pessoa com Deficiência (CEPRED), Salvador, Brazil; ^3^ Departamento de Psicologia, Universidade do Estado da Bahia, Salvador, Brazil; ^4^ Departamento de Neurociências e Saúde Mental, Faculdade de Medicina da Bahia, Universidade Federal da Bahia, Salvador, Brasil

**Keywords:** Congenital Zika Syndrome, developmental disabilities, cerebral palsy, abnormal reflex, motor skills, abnormal posturing

## Abstract

**Background:**

The Zika virus outbreak, which occurred from 2015 to 2016 in Brazil, resulted in the birth of neonates with brain malformations arising from Congenital Zika Syndrome (CZS). The characterization of primitive reflexes and their relationships with other motor characteristics, easily clinically detectable by health professionals, can aid in establishing motor prognosis in affected children.

**Objective:**

To describe reflex patterns in children with Cerebral Palsy (CP) due to CZS, and investigate associations with other motor features. Method: Observational cross-sectional study involving infants with CZS aged between 12 and 36 months. Primitive reflexes, protective reaction and markers of motor phenotype were evaluated.

**Results:**

48 children, median age: 19 months, were enrolled, most (79.2%) presented very severe CP (GMFCS 5), the persistence of more than 5 primitive reflexes (55%) and motor development age between 3 and 6 months (33.3%) (Bayley-III). A reduced ability to acquire motor skills was associated with the total number of persistent reflexes (rho = −0.45, *p* < 0.01). Asymmetrical Tonic Neck Reflex (ATNR) correlated with GMFCS level (rho = 0.49, *p* < 0.001). Lower motor development age was linked to abnormal posturing (*p* < 0.001) and absence of Parachute Reaction (*p* < 0.001).

**Conclusion:**

Infants with CP due to CZS present severe motor abnormalities. Lower motor development age is associated with the persistence of more than 5 primitive reflexes, abnormal posturing and the absence of Parachute Reaction. Parachute Reaction appears to be a prognostic marker of motor impairment severity in CZS-affected infants.

## Introduction

The Zika virus (ZIKV) is an emergent flavivirus transmitted by the *Aedes* mosquitoes (1). In 2015, ZIKV transmission was confirmed in Brazil (2), resulting in a large-scale epidemic that later spread to other countries throughout the Americas. In late 2015, an unexpected outbreak of newborns with microcephaly prompted a state of public health emergency in Brazil. The ZIKV maternal-fetal transmission was confirmed, and until 2020, 3,563 cases of Congenital Zika Infection (CZI) were confirmed (3).

CZI can result in a spectrum of clinical manifestations, including asymptomatic cases (4). Congenital Zika Syndrome (CZS) is characterized by neurodevelopmental delays, microcephaly, and severe Cerebral Palsy (CP) (5, 6). Severe motor delay, persistence of primitive reflexes, and postural abnormalities have been described (7–11). However, the literature contains no studies associating the responses of primitive reflexes in children with CVS with other phenotypic markers. The present study aimed to investigate reflex responses in infants with CP arising from CZS, and explore their associations with other phenotypic markers.

## Methods

### Study design and participants

The present observational, cross-sectional study evaluated reflex responses and motor characteristics in infants with CZS presenting CP. Study participants were recruited at the Center for Prevention and Rehabilitation of Disabilities of the State of Bahia (CEPRED), located in the city of Salvador (Bahia, Brazil). The CEPRED is the state reference center for follow-up of children with CZS in the local public health system.

Infant age ranged between 12 and 36 months, including both sexes, born during the 2015–2016 outbreak of the Zika virus in Brazil. The inclusion criteria consisted of CZS, either confirmed by ZIKV test positivity or not, as well as clinical findings including neurological malformations and/or specific neuroimaging findings (12). All other congenital infections were excluded. Children presenting arthrogryposis were also excluded, as this condition could interfere with the results obtained from the assessment.

Microcephaly was defined according to the International Fetal and Newborn Growth consortium for the 21st Century (Intergrowth-21) criteria. Microcephaly was considered when the head circumference at birth measured less than two standard deviations below the average, while severe microcephaly was determined when measurements were less than three standard deviations below the average (13).

### Assessments and procedures

Motor function testing and reflex responses were assessed via clinical and instrumental analysis. Clinical evaluations included the inspection of the infant's posture and movements (14–16). Postural symmetry was considered when an imaginary line through the shoulders and hip joints runs parallel (14). Abnormal tonic posturing was considered when an infant was placed in a supine position and was unable to actively move their limbs, remaining tonically fixed in an abnormal posture.

Gross Motor Subtest raw scores, derived from the Bayley-III Scales of Infant and Toddler Development, were used to estimate the developmental age of child motor development. The Bayley III is a standardized and specific instrument for evaluating cognitive, language, and motor development (17, 18). The Gross Motor Function Classification System (GMFCS) is a five-level framework designed to describe and categorize the severity of motor impairments in children with cerebral palsy (CP) and it has been utilized to classify the motor function of individuals with CP. Level I represents the best gross motor abilities (CP children and youth who walk without limitations), and level V represents the poorest function (children who require a wheelchair) (19). Finally, a checklist was elaborated to evaluate CP topography, global tone, posture and active mobility, as well as reflexes, protective reaction and motor skills (15–21).

The following reflexes were evaluated: Moro Reflex, Asymmetrical Cervical Tonic Reflex, Symmetrical Cervical Tonic Reflex, Labyrinthine Tonic Reflex, Palmar Grasp, Plantar Grasp, Positive Support Reflex, Gait Reflex, Cross Extension Reflex. The Parachute Reaction was employed to assess protective reaction (15, 16, 22). Based on tools and studies (17, 20, 22), the following acquired motor skills were evaluated: complete/incomplete cervical control, partial/total rolling, sitting with/without support, crawling movement, transition from supine to sitting position, standing from sitting position, standing with/without support, walking with/without support, maintenance of crawling position, crawling on stomach.

### Procedures

The evaluation sequence was similar for all children, starting with a visual inspection, in the supine position, of posture, CP topography and active movements. Next, the Bayley-III and GMFCS scales were applied. Finally, motor skills, reflexes and protective reaction were evaluated. All children were evaluated by the same trained physical therapist with experience in infants with CZS.

### Data management and statistical analysis

Data entry and management were performed using REDCap 9.3.1 software (© 2021 Vanderbilt University). Statistical analysis was performed using SPSS v21.0 software for Windows. Descriptive statistics were calculated for the clinical and motor parameters evaluated. Student's T test was used to determine differences between mean values of the number of motor skills and the presence of Parachute Reaction and Abnormal Posturing. Also, for Motor Development Age and presence of Parachute Reaction. Spearman's correlation coefficient was used to determine associations between the number of motor skills acquired and Palmar Grip Reflex response grade, Motor Development Age and total number of Persistent Reflexes. Simple linear regression was applied to verify correlations between Motor Development Age and the presence of Parachute Reaction, Intergrowth and total number of Primitive Reflexes. Statistical significance was considered when *p* < 0.05.

### Ethical considerations

The present study was approved by the Institutional Review Board of the Gonçalo Muniz Institute, Oswaldo Cruz Foundation (IGM-FIOCRUZ, protocol no. 1.935.854/2016). The legal guardians of all infants provided written informed consent.

## Results

Eighty-five children were recruited, 20 of whom did not attend their scheduled evaluations, six were excluded due to arthrogryposis and two did not complete the entire assessment. In all, 48 infants were enrolled.

The maternal socioeconomic profile is detailed in Table 1. The majority of the mothers self-identified as Black or mixed-race, and their household monthly income was predominantly low, with a mean of 1.3 ± 0.6 Brazilian minimum wages.

**Table 1:** Clinical and demographical features of 48 infants with Congenital Zika Syndrome (Salvador, Bahia-Brazil).

Features	*n* = 48 (%)
Self-reported Maternal Race/Ethnicity	
Mixed race	17/37 (45,9%)
Black	17/37 (45,9%)
White	3/37 (8,1%)
Maternal Years of Education	
0–8 years	4/21 (18,9%)
9–12 years	13/21 (61,8%)
≥13 years	4/21 (18,9%)
Household monthly Income in Minimum Wage^a^ (mean ± SD)	1,3 ± 0,6
Age in months (median ± Q_3_-Q_1_)	19 (26.2–15.2)
Sex	
Male	21/48 (43,7)
Female	27/48 (56.2)
Gestational Age in weeks (mean ± SD)	38.5 ± 1.7
Prematurity	5 (10.4)
Weight at birth (grams) (mean ± SD)	2,753.4 ± 509
Length at birth (cm) (mean ± SD)	45.2 ± 3
Head circumference at birth (cm) (mean ± SD)	29.3 ± 2.2
Intergrowth	
Normal (0 and −1)	11/48 (23.4)
Microcephaly (−2)	14/48 (29.8)
Severe Microcephaly (≤ −3)	22/48 (46.8)
Neuroimaging abnormalities (*n* = 41)	
Calcifications	41/41 (100)
Ventricular enlargement	34/41 (82.9)
Other findings (lissencephaly, volumetric reduction, agenesis of the corpus callosum, hydrocephalus)	17/41 (41.4)
Epilepsy	34/48 (70.8)

^a^ Based on monthly minimum wage (R$ 1,412.00, equivalent to US$ 232.25).

The median age of the infants was 19 (26.2–15.5) months; 56.2% were female. Mean gestational age at birth was 38 ± 1.7 weeks, with prematurity identified in 10.4%. Regarding head circumference at birth, 46.8% presented severe microcephaly (Intergrowth ≤−3). The clinical and demographical characteristics of the participants are shown in Table 1.

**Figure 1:**
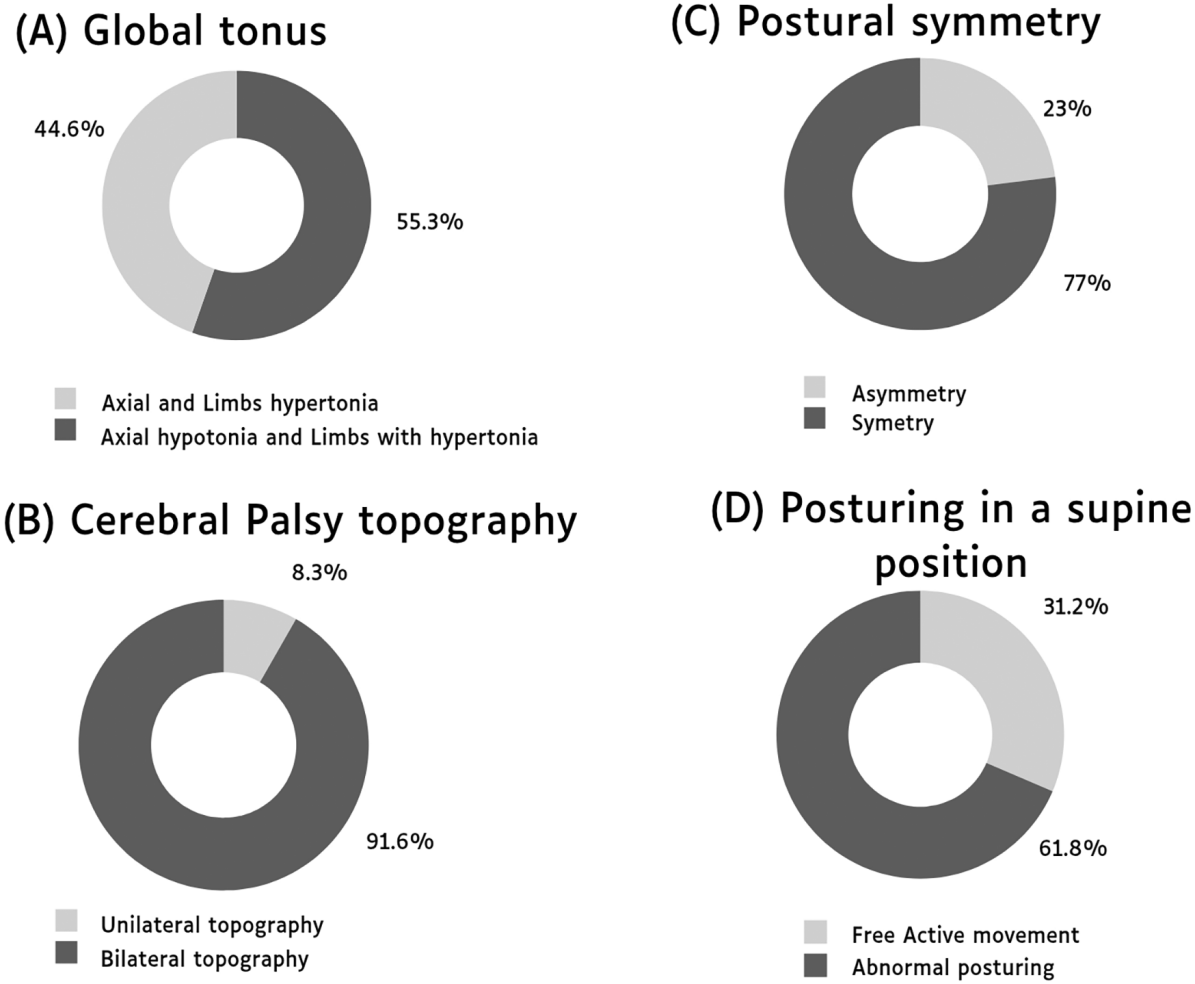
Distribution of key motor characteristics in individuals with cerebral palsy due to Congenital Zika Syndrome. **(A)** Global tonus. **(B)** Cerebral palsy topography. **(C)** Postural symmetry. **(D)** Posturing in a supine position.

The main features observed were axial hypotonia with hypertonic limbs, symmetric posture, bilateral CP topography, and abnormal posturing in a supine position and few children without movement restrictions. The motor characteristics of the studied infants are shown in Figure 1.

Twenty-two children (45.8%) had no motor skill acquisition, followed by 14 (29.2%) with at least incomplete cervical control. There was a predominance of children with GMFCS level 5 and motor age between 3 and 6 months. The acquisition of motor skills in these children is detailed in Table 2. The Parachute Reaction was observed in nine (19.1%) infants, with detailed information on Primitive Reflexes listed in Table 3.

**Table 2:** Motor development characteristics in infants with Congenital Zika Syndrome, (Salvador, Bahia-Brazil).

Motor development characteristics	*f* (%)
Motor skills	*N* *=* *48 (%)*
No acquisition	22 (45.8)
Cervical control	
Incomplete	12 (25)
Complete	14 (29.2)
Rolling	
Partial	13 (27.1)
Total	10 (20.8)
Sitting	
With the support of members	9 (18.8)
Without the support of members	5 (10.4)
Dragging	7 (14.6)
Crawling position	1 (2.1)
Crawling movement	1 (2.1)
Supine to sitting	2 (4.2)
Sitting to standing	2 (4.2)
Standing with support	3 (6.3)
Walking with support	2 (4.2)
Walking without support	1 (2.1)
GMFCS	
1 e 2	2/47 (4.2)
3	0/47 (0)
4	8/47 (16.7)
5	38/47 (79.2)
Bayley-III scale	
Development motor age	
0–15 days	10/48 (20.8)
16 days to 1 month	3/48 (6.2)
1 to 3 months	11/48 (22.9)
3 to 6 months	16/48 (33.3)
6 to 9 months	7/48 (14.5)
More than 9 months	1/48 (2)

GMFCS,  Gross Motor Function Classification System; f, frequency.

**Table 3:** Frequency of persistent primitive reflexes and types of abnormal posturing in infants with Congenital Zika Syndrome (Salvador, Bahia-Brazil).

Primitive reflexes	*f* (%)
Persistence of primitive reflexes	
No persistence of primitive reflexes	1 (2.1)
1 persistent reflex	2 (4)
2–4 persistent reflexes	19 (41)
>5 persistent reflexes	25 (55)
Persistence reflexes found	
Moro reflex	12 (25.5)
ATNR (Asymmetrical Tonic Neck Reflex)	24 (51)
STNR (Symmetrical Tonic Neck Reflex)	14 (29.7)
TLR (Tonic Labyrinthine Reflex)	14 (29.7)
Palmar grip reflex	33 (70.2)
Plantar grasp reflex	34 (72.3)
Positive support reflex	30 (63.8)
Automatic gait reflex	16 (34)
Crossed extensor reflex	26 (55.3)
Abnormal posturing types	*f* (%)
Fixed posture- extended upper limbs and flexed lower limbs	1/47 (2.1)
Fixed posture- backward arching of the head and body (opisthotonus)	1/47 (2.1)
Fixed posture- Asymmetric posture	2/47 (4.2)
Fixed posture- Flexed upper limbs with extended lower limbs	12/47 (25.5)
Fixed posture- Batrachian posture	13/47 (27.6)

### Primitive reflexes and motor skills

The lack of ability to acquire motor skills was correlated with higher grades of Palmar Grip Reflex responses (Left rho = −0.37, *p* < 0.01; Right rho = −0.45, *p* < 0.05), and the total number of persistent reflexes (rho = −0.45, *p* < 0.01). The presence of the Moro Reflex did not seem to significantly affect the number of motor skills acquired.

### Primitive reflexes and CP

The presence of Asymmetrical Tonic Neck Reflex (ATNR) was found to be correlated with GMFCS classification (rho = 0.49, *p* < 0.001).

### Motor development age, abnormal posturing and parachute reaction

We identified a correlation between Motor Development Age and the Number of Persistent Reflexes (rho = −0.3, *p* < 0.05). In addition, abnormal posturing was significantly associated (*p* < 0.001) with Motor Development Age, as a lower average development age was observed in the presence of abnormal posturing.

A younger Motor Development Age was also associated with the absence of the Parachute Reaction (*p* < 0.001). Accordingly, the presence of the Parachute Reaction was identified as a predictor of greater Motor Development Age (*β*=0.743; *p* < 0.001). By contrast, Intergrowth measures offered lower predictive power of Motor Development Age (*β*=0.407; *p* < 0.001).

## Discussion

The study was conducted at a public rehabilitation center in Bahia, Brazil, one of the regions most severely impacted by the Zika epidemic. The maternal socioeconomic profile observed in this study aligns with findings from other Brazilian studies involving pregnant women infected with ZIKV (23, 24).

The present study investigated reflex patterns in infants with CP due to CZS, as well as motor characteristics. In children with severe CP, marked impairment of global motricity was observed. In addition, these children also presented the persistence of more than five primitive reflexes, different presentations of abnormal posturing in the supine position, and impaired limb mobility and motor development. A very low age of motor development was also noted, as well as a low number of acquisitions of motor milestones, e.g., the ability to maintain sitting or standing positions. The presence of hypotonia in combination with hypertonia was also observed, similar to another previous study (25).

Other studies have previously demonstrated the persistence of primitive reflexes in children with CZS (8, 9, 10, 26, 27). A study by Armani et al. (28) evaluated six reflexes, with persistence identified in 56% of the participants. Our study aimed to expand the scope of reflex evaluations by testing nine reflexes, with persistence observed in 97.8% of the infants studied.

Several presentations of abnormal posturing were observed in 68.1% of our sample. Other studies on CZS in infants have reported the presence of abnormal posturing, such as axial extensor postures or flexor postures (6), in 74.7% of the population studied, while dystonic postures (8) were present in 95.2% of infants. Hull, Parnes and Jankovic (29) suggested that abnormal posturing, such as opisthotonos, is often associated with dystonic posturing of the limbs. While it is possible that an association between dystonia and abnormal posturing in our population, this aspect was not investigated.

We identified a correlation between the persistence of primitive reflexes and lower number of acquired Motor Skills, younger Motor Development Age, and greater CP severity. Despite a high prevalence of primitive reflexes, the presence of abnormal posturing appeared to be strongly associated with younger Motor Development Age. It is possible that, when lying in a supine position, exacerbated basal tonus restrains the free movement of the body, severely limiting motor acquisition, as was suggested by Van der linden et al. (8), who argued that the severity and quality of dystonic postures, among other factors, can vary in accordance with body position. Abnormal posturing, when present, provokes discomfort, and its consequences may lead to contractures and deformities (29). On the other hand, it is possible that while primitive reflexes may be observed when elicited by a certain stimulus, these are not necessarily the only factors underlying the inhibition of global motricity. More studies are needed to fully elucidate the mechanisms involved in motor behavior in the context of CZS.

The Parachute Reaction is known to offer predictive value in CP after nine months of age (30). Herein, all of the enrolled infants exhibited some degree of CP. Our findings indicate that, in the children with CP due to CZS who were 12 months or older, the absence of a parachute reaction may constitute a prognostic marker of younger Motor Development Age. While other studies have demonstrated associations between Intergrowth score and motor development (31, 32), as was observed herein, our results suggest a correlation between the Parachute Reaction and higher Motor Development Age.

This study aimed to comprehensively detail reflex patterns and establish associations with other relevant clinical characteristics. Our findings demonstrate that infants with CP due to CZS exhibit severe motor abnormalities, abnormal posturing, and the persistence of primitive reflexes, all of which are associated with impaired motor development. Parachute Reaction appears to be a prognostic marker of motor impairment severity in CZS-affected infants.

A selection bias may exist, since severe CP presentations predominated in our sample, as the study was carried out at a Reference Rehabilitation Service specializing in auditory, physical and mental impairment. Children with milder presentations may not have been referred to the rehabilitation center. Indeed, herein, this fact proved to be advantageous, offering the possibility to describe as extensively as possible the characteristics of CZS-associated impairment in these children, similarly to other studies conducted in different regions of Brazil (7, 8, 9, 25). Moreover, further studies are needed to clarify the role of the Parachute Reaction and to validate its use as a clinical marker for motor prognosis in this population. Additionally, research involving children with less severe CZI is necessary, as the majority of participants in this study had CP with severe disabilities, classified as GMFCS levels IV and V.

Given the widespread presence of *Aedes* mosquitoes and the ongoing threat of future ZIKV outbreaks (33), the findings presented here provide valuable insights and could contribute to the management and monitoring of newly affected children.

## Data Availability

The raw data supporting the conclusions of this article will be made available by the authors, without undue reservation.
